# Development and immunogenicity evaluation of a quadruple-gene-deleted pseudorabies virus strain

**DOI:** 10.3389/fmicb.2024.1479794

**Published:** 2024-09-20

**Authors:** Hui Li, Riteng Zhang, Jiahao Qu, Yahao Kang, Jingnan Zhang, Ruhai Guo, JunDa Li, Xiao Zhang, Likang Han, Honglin Xie, Xinglong Wang

**Affiliations:** ^1^College of Veterinary Medicine, Northwest A&F University, Yangling, China; ^2^College of Veterinary Medicine, Gansu Agricultural University, Anning, China; ^3^School of Animal Science and Technology, Foshan University, Foshan, China

**Keywords:** pseudorabies virus, recombinant vaccine, gene deletion, CRISPR/Cas9, immunogenicity

## Abstract

Since 2011, the emergence of Pseudorabies virus (PRV) variants has led to significant vaccine failures, resulting in severe economic losses in China’s swine industry. Conventional PRV vaccines have shown limited efficacy against these emergent variants, underscoring the urgent need for novel immunization strategies. This study aimed to develop and evaluate a novel recombinant PRV vaccine candidate with improved safety and immunogenicity profiles. Utilizing the homology-directed repair (HDR)-CRISPR/Cas9 system, we generated a recombinant PRV strain, designated PRV SX-10ΔgI/gE/TK/UL24, with deletions in the gI, gE, TK, and UL24 genes. *In vitro* analyses demonstrated that the recombinant virus exhibited similar replication kinetics and growth curves comparable to the parental strain. The immunological properties of the recombinant PRV were assessed in murine and porcine models. All animals inoculated with PRV SX-10ΔgI/gE/TK/UL24 survived without exhibiting significant clinical signs or pathological alterations. Immunological assays revealed that PRV SX-10ΔgI/gE/TK/UL24 elicited significantly higher levels of gB-specific antibodies, neutralizing antibodies, and cytokines (including IFN-*γ*, IL-2, and IL-4) compared to both the Bartha-K61 and PRV SX-10ΔgI/gE/TK strains. Notably, both murine and porcine subjects immunized with PRV SX-10ΔgI/gE/TK/UL24 demonstrated enhanced protection against challenges with the variant PRV SX-10 strain, compared to other vaccine strains. These findings suggest that PRV SX-10ΔgI/gE/TK/UL24 represents a promising PRV vaccine candidate strain, offering valuable insights for the prevention and control of PRV in clinical applications.

## Introduction

Pseudorabies (PR), also known as Aujeszky’s disease (AD), is an acute and highly contagious viral infection caused by the pseudorabies virus (PRV), a member of the Herpesviridae family (Mettenleiter, 2000). PRV infects a wide range of mammals, including swine, rodents, canines, bovines, ovines, and lagomorphs, with pigs serving as the primary host and reservoir (Fenner et al., 1987; Pensaert and Kluge, 1989; Sun et al., 2022). The disease manifestation varies among susceptible species, but in swine, it can lead to severe economic losses due to neurological disorders, respiratory distress, and weight loss in newborn piglets, as well as high mortality rates in young piglets and abortions in pregnant sows (Pomeranz et al., 2005; Sun et al., 2016). Additionally, PRV infection in humans has been associated with endophthalmitis and encephalitis (Liu et al., 2020; Yang et al., 2019; Zheng et al., 2022). PRV was first reported in China in 1947 and subsequently became endemic across most regions of the country (Tong and Chen, 1999). In the 1990s, the introduction and widespread application of the gene-deleted Bartha-K61 vaccine from Hungary significantly contributed to controlling the PR epidemic in China (Freuling et al., 2017; Wang et al., 2014; Wu et al., 2013; Zhou et al., 2017). However, since 2011, emerging PRV variants have demonstrated potentially enhanced pathogenicity compared to classical strains, particularly in piglets (An et al., 2013; Hu et al., 2015; Lian et al., 2023; Tong et al., 2015; Ye et al., 2016; Yu et al., 2014). Notably, the outbreaks of PR have occurred even in pig herds immunized with the Bartha-K61 vaccine, indicating that traditional PRV vaccines May be ineffective against these emerging variant strains (Cong et al., 2016; Luo et al., 2014; Wang et al., 2019). These emerging challenges underscore the critical need for developing safer and more efficacious vaccines to combat the evolving PRV strains and mitigate their potential long-term economic impact on the swine industry.

Extensive research on the UL24 gene of herpesviruses has revealed that while the UL24 protein is not essential for viral replication *in vitro* (Jacobson et al., 1998; Whitbeck et al., 1994), but its deletion attenuates viral virulence (Blakeney et al., 2005; Jacobson et al., 1998; Kasem et al., 2010; Rochette et al., 2015; Sanabria-Solano et al., 2016). Studies in various herpesvirus models have consistently demonstrated this attenuation effect: In HSV-2, UL24 gene deletion markedly reduced viral pathogenicity in murine and guinea pig models compared to wild-type strains (Blakeney et al., 2005; Leiva-Torres et al., 2010). An EHV-1 strain lacking ORF37 (a homologous gene) exhibited no neurotoxicity or lethality in a murine model (Kasem et al., 2010). Additionally, UL24-deficient HSV-1 infections resulted in significantly lower viral titers in murine trigeminal ganglia (Jacobson et al., 1998; Rochette et al., 2015). This characteristic is pivotal for the development of efficacious attenuated vaccines. Notably, recent investigations have elucidated the involvement of UL24 in viral pathogenesis. In PRV, UL24 has been shown to interact with IRF7 through the proteasome pathway, thereby antagonizing cGAS-STING-mediated IFN-*β* activity (Liu et al., 2021). This interaction significantly contributes to viral immune evasion during host-pathogen interactions (Ruan et al., 2023), suggesting that UL24 is a crucial virulence factor in PRV, inhibiting the host’s innate immune response. Furthermore, extensive research has demonstrated that the deletion of gI, gE, and TK genes significantly attenuates PRV virulence without substantially impacting the virus’s immunogenicity, identifying these as critical targets for the development of safe vaccines (Lei et al., 2016; Zhao et al., 2020). Given these findings, targeting UL24 for the construction of an attenuated vaccine holds strategic significance. This approach could potentially yield a vaccine that maintains low virulence while effectively stimulating the immune system, thus addressing the critical need for safer and more efficacious PRV vaccines.

In this study, we employed the CRISPR/Cas9 system to engineer a recombinant PRV variant strain deficient in gI, gE, TK, and UL24 genes, aiming to develop a vaccine strain with enhanced immunogenicity while maintaining attenuated virulence. A comprehensive evaluation of the safety profile and immunogenic properties of PRV SX-10ΔgI/gE/TK/UL24 was conducted in both murine models and weaned porcine subjects to assess its potential as a vaccine candidate. The findings from this investigation provide a crucial foundation for the development of efficacious vaccines against emergent PRV variant strains.

## Results

### Generation of recombinant PRV with deletions in gI/gE/TK/UL24 or UL24 via CRISPR/Cas9 system

**Figure 1:**
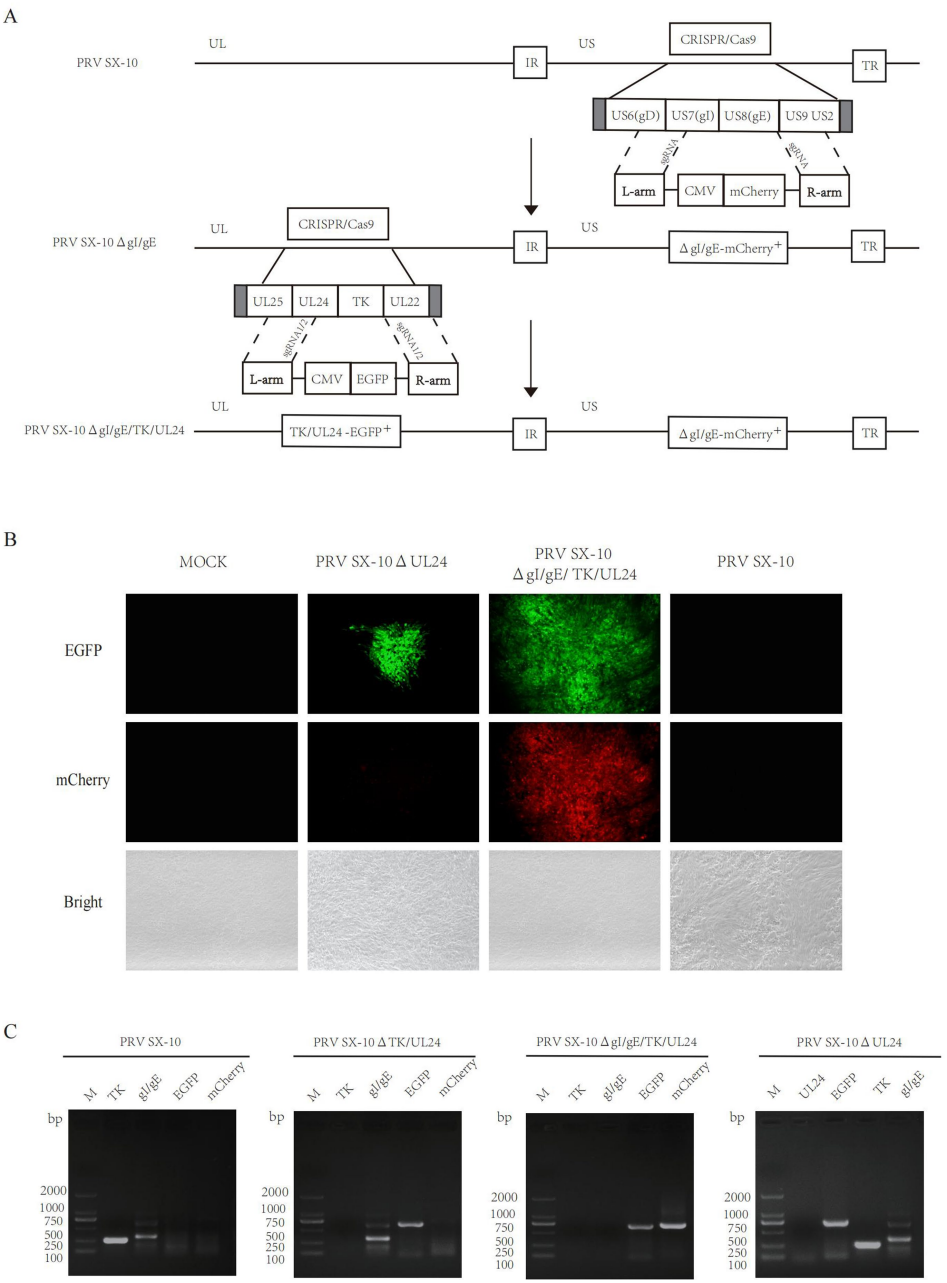
Construction of recombinant viruses PRV SX-10ΔgI/gE/TK/UL24 and PRV SX-10ΔUL24. **(A,B)** Schematic diagram of recombinant virus strain construction and plaque purification. **(C)** PCR identification of TK, gI/gE deletion and EGFP, mCherry insertion in recombinant viruses.

The UL24 gene of PRV has been confirmed to play a pivotal role in regulating innate immune responses in host cells (Liu et al., 2021; Wang et al., 2020). Here, a variant PRV SX-10 virus lacking the gI, gE, TK, and UL24 genes was generated using the CRISPR/Cas9 technology, as shown in Figure 1A. Additionally, a similar procedure was utilized to generate a mutant PRV SX-10 virus lacking only the UL24 gene, named PRV SX-10ΔUL24 (Figure 1B). The purified viruses were confirmed via PCR assays with specific primers to the viral gI/gE, TK, UL24, EGFP, and mCherry (Figure 1C).

### Growth characteristics in diverse PRV strains

**Figure 2:**
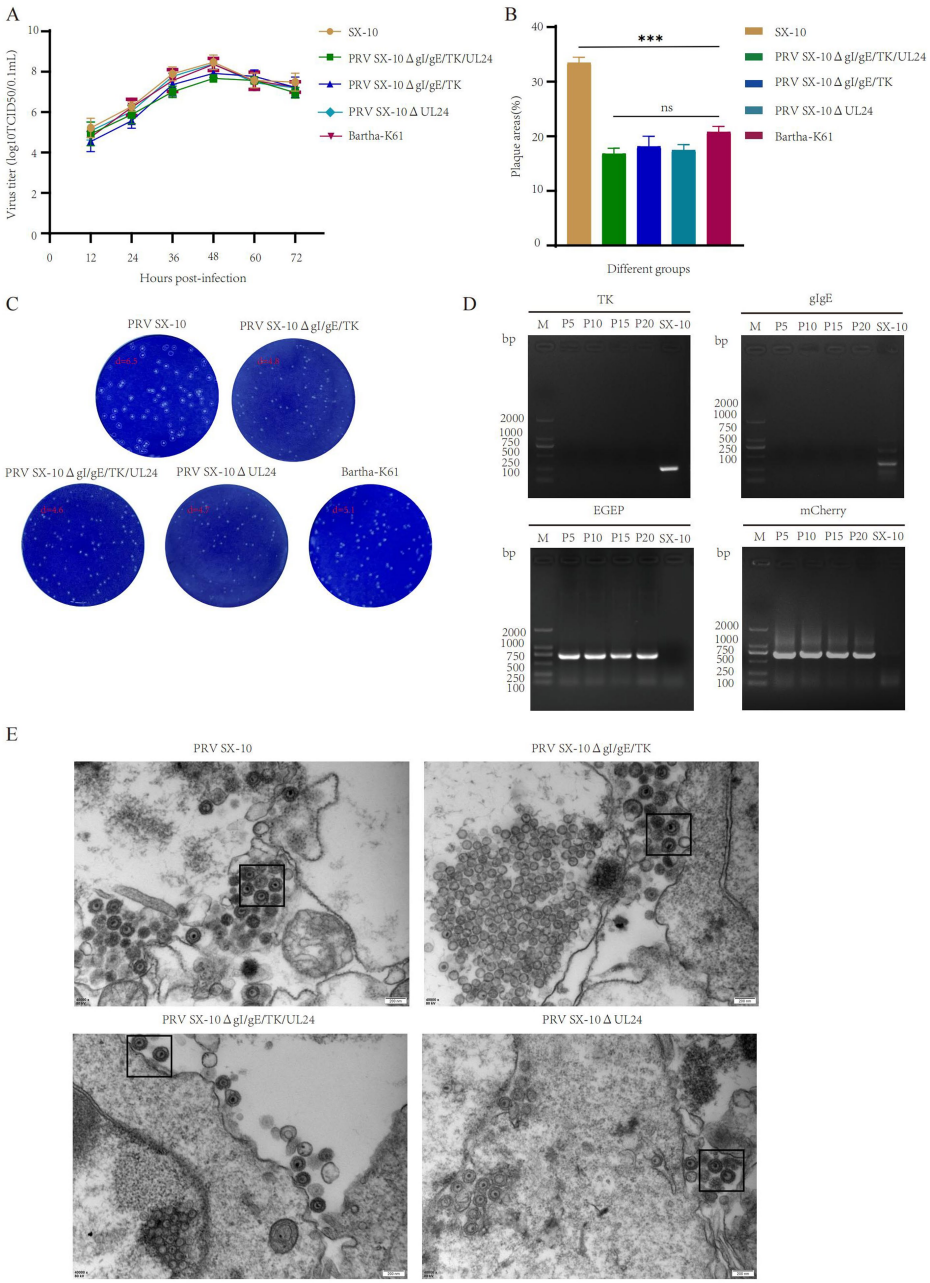
Study on the biological characteristics of different PRV virus strains. **(A)** Growth curves of the parental strain PRV SX-10, and the recombinant virus strains PRV SX-10ΔgI/gE/TK, PRV SX-10ΔgI/gE/TK/UL24, PRV SX-10ΔUL24, and Bartha-K61. **(B,C)** Measure plaque size and morphology of different virus strains and parental strain PRV SX-10. The plaque areas were counted and calculated using ImageJ software. ***p < 0.001, *ns*: not significant. **(D)** PCR identification of TK and gI/gE deletions and EGFP and mCherry insertions in the recombinant virus strain PRV SX-10ΔgI/gE/TK/UL24, with the parental strain PRV SX-10 as a control. **(E)** Transmission electron microscopy analysis of PK-15 cells infected with recombinant and parental strains.

The peak virus titers of PRV SX-10ΔgI/gE/TK/UL24, PRV SX-10ΔgI/gE/TK, PRV SX-10ΔUL24, Bartha-K61, and wild-type PRV SX-10 were observed at 48 h post-infection, reaching 10^7.67^, 10^7.92^, 10^8.39^, 10^8.36^, and 10^8.47^ TCID_50_/mL, respectively. Moreover, the one-step growth curves were similar among the recombinant PRVs and PRV SX-10 (Figure 2A). Compared to the wild-type PRV SX-10 strain, the plaque sizes formed by the recombinant virus strains and Bartha-K61 were significantly smaller (Figures 2B,C). PRV SX-10ΔgI/gE/TK/UL24 was serially passaged in PK-15 cells for 21 generations (F21), and the relevant genes were verified using PCR analysis (Figure 2D). Transmission electron microscopy analysis revealed that the morphology of viral particles from recombinant virus strains was indistinguishable from that of wild-type PRV SX-10, with no discernible structural differences observed (Figure 2E).

### Deletion of UL24 enhances IFN-β expression and reduces virulence

**Figure 3:**
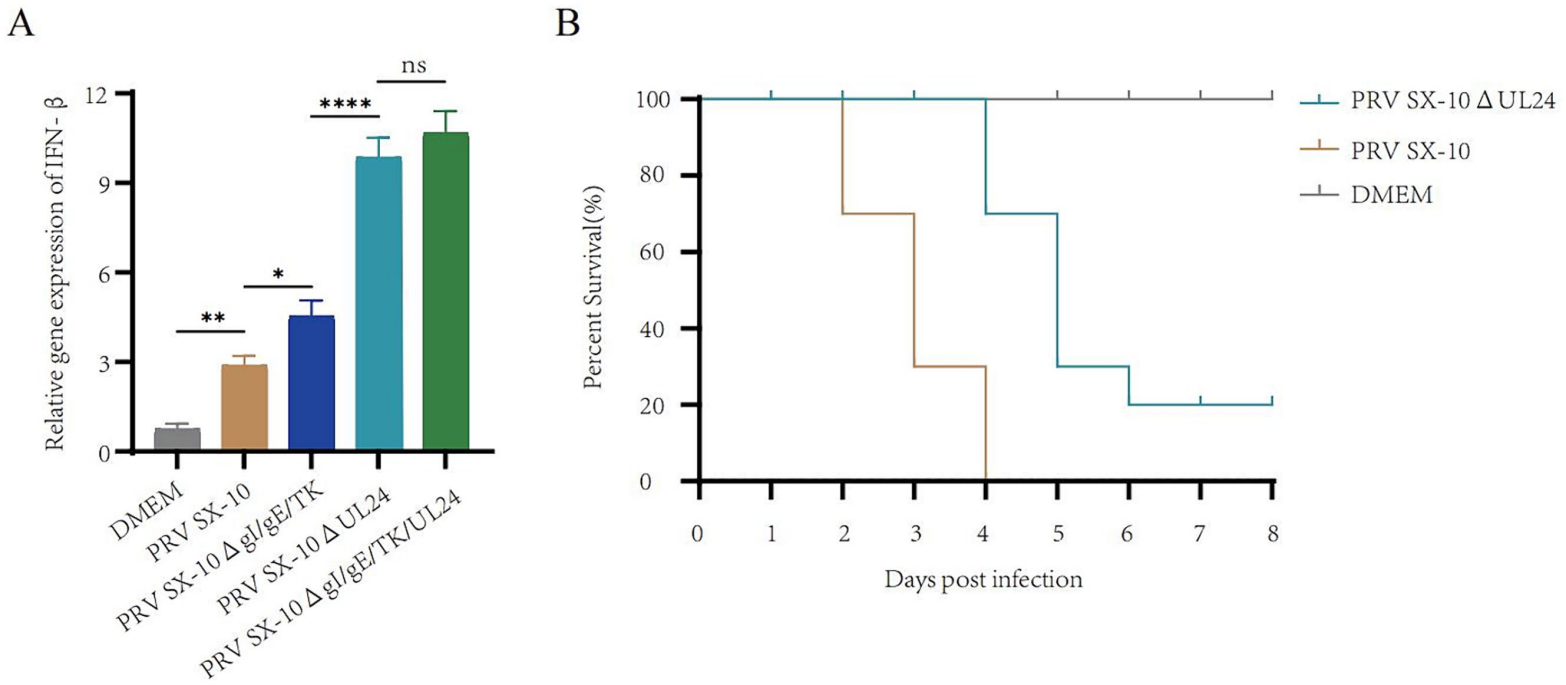
Study on the relevant characteristics of the virus strain PRV SX-10ΔUL24. **(A)** PK-15 cells were inoculated with PRV SX-10, PRV SX-10ΔgI/gE/TK, PRV SX-10ΔUL24, and PRV SX-10ΔgI/gE/TK/UL24 virus strains (MOI = 0.1) for 12 h, mRNA level was measured by RT-qPCR. ***p < 0.001, and *ns* as not significant. **(B)** Survival curves of mice infected with PRV SX-10 and PRV SX-10ΔUL24 strains, DMEM as a control group.

The UL24 of herpesviruses suppress IFN-β and reduce pathogenicity in mice (Jacobson et al., 1998; Liu et al., 2021). Real-time PCR analysis revealed that IFN-β transcripts levels were significantly elevated in cells infected with PRV SX-10ΔUL24 and PRV SX-10ΔgI/gE/TK/UL24 compared to those infected with PRV SX-10 or PRV SX-10ΔgI/gE/TK strains, or treated with DMEM (Figure 3A). Moreover, in the mouse challenge experiment, mice infected with PRV SX-10ΔUL24 exhibited a 20% higher survival rate compared to those challenged with wild-type PRV SX-10 (Figure 3B).

### Determination of immune characteristics of recombinant PRV SX-10ΔgI/gE/TK/UL24 in mice

**Figure 4:**
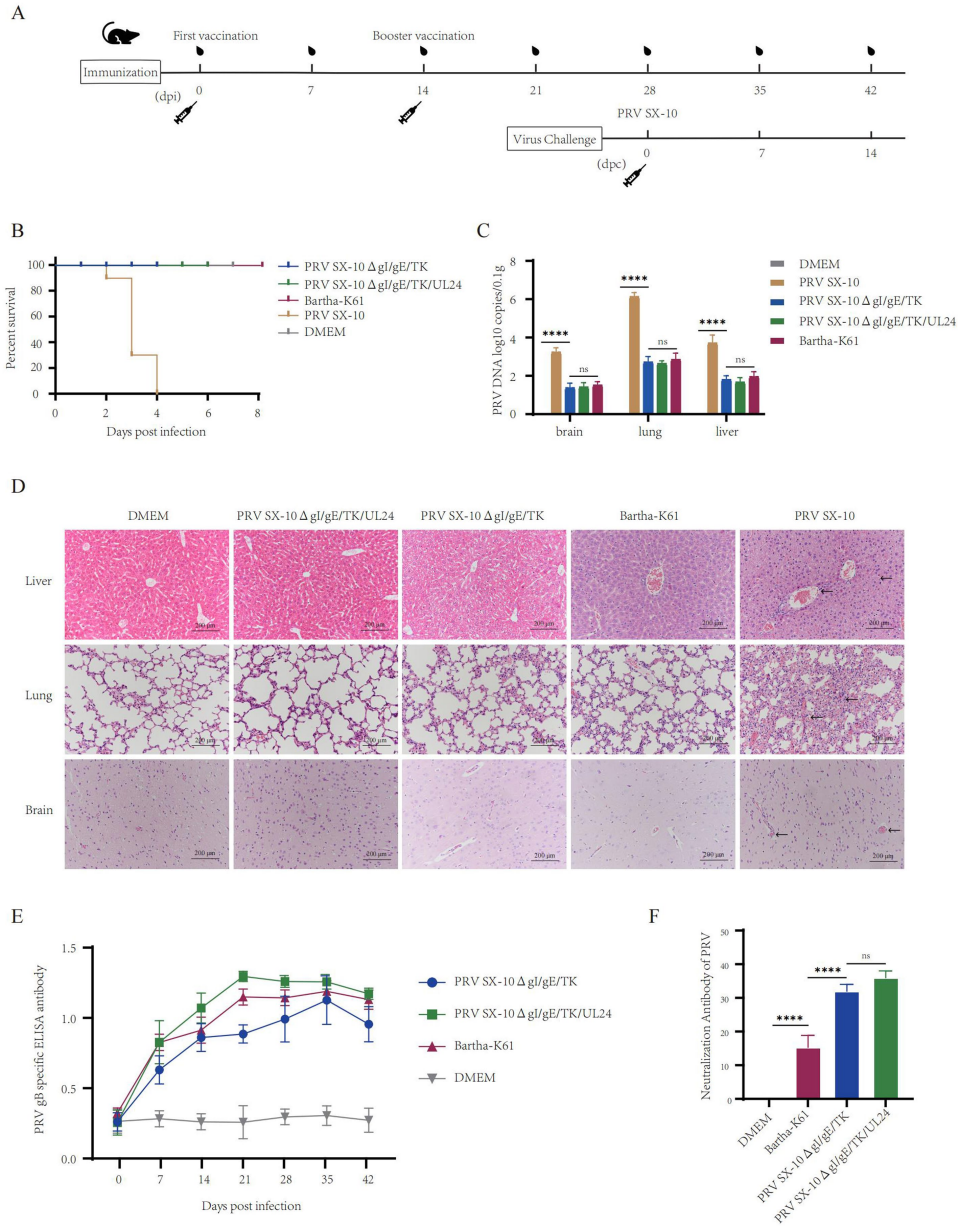
Safety and immunogenicity evaluation of recombinant virus in mice. **(A)** Schematic diagram of the timeline for mouse immunization and challenge. **(B)** Survival rate of mice immunized with recombinant virus strains and challenged with the PRV SX-10 strain, DMEM as a control group. **(C)** Detection of viral loads in the brain, lungs, and liver after immunization using qPCR. ****p < 0.0001, and *ns* as not significant. **(D)** Histopathological analysis of liver, lung, and brain from each group after immunization (HE, 200×). Arrows in the PRV SX-10 group indicate locations of lesions. **(E)** Detection of gB antibodies at designated time points after immunization. Mouse serum samples with ELISA values ≥ 0.38 were considered positive and <0.2 were considered negative. **(F)** Determination of virus-neutralizing antibody titers in serum collected 28 dpi. **p < 0.01, and *ns* as not significant.

The safety and immunogenicity of the recombinant viruses were evaluated in mice (Figure 4A). Mice inoculated with 100 μL containing 10^5^ TCID₅₀/mL of PRV SX-10ΔgI/gE/TK/UL24, PRV SX-10ΔgI/gE/TK, Bartha-K61, or DMEM (control group) all survived without clinical symptoms through 14 days post-immunization (dpi). However, mice inoculated with PRV SX-10 exhibited typical pseudorabies (PR) symptoms, including severe pruritus, ruffled fur, and hyperkinesia, with all subjects succumbing to the infection within 2–4 days post-inoculation (Figure 4B). At 14 dpi, a subset of mice was euthanized and subjected to necropsy. The viral loads in tissues from mice in the PRV SX-10ΔgI/gE/TK/UL24 groups were significantly lower than those in the PRV SX-10 group (Figure 4C). Compared to the DMEM control group, brain, liver, and lung tissues from mice challenged with PRV SX-10ΔgI/gE/TK/UL24, PRV SX-10ΔgI/gE/TK, or Bartha-K61 strains exhibited no significant histopathological changes. In contrast, mice infected with PRV SX-10 exhibited cerebral vascular congestion and perivascular lymphocytic cuffing in brain tissues, pulmonary congestion and edema in the lungs, and congestion in the liver (Figure 4D). Additionally, the PRV SX-10ΔgI/gE/TK/UL24 vaccinated group exhibited a significantly stronger antibody response compared to groups vaccinated with PRV SX-10ΔgI/gE/TK, especially on 21 dpi (*p* < 0.0001) (Figure 4E). Concurrently, the PRV SX-10ΔgI/gE/TK/UL24 immunized group and the PRV SX-10ΔgI/gE/TK immunized group exhibited similar neutralization titers at 28 dpi, which were significantly higher than those of the Bartha-K61 immunized group (*p* < 0.0001) (Figure 4F).

### Evaluation of protective efficacy of PRV SX-10ΔgI/gE/TK/UL24 in mice

**Figure 5:**
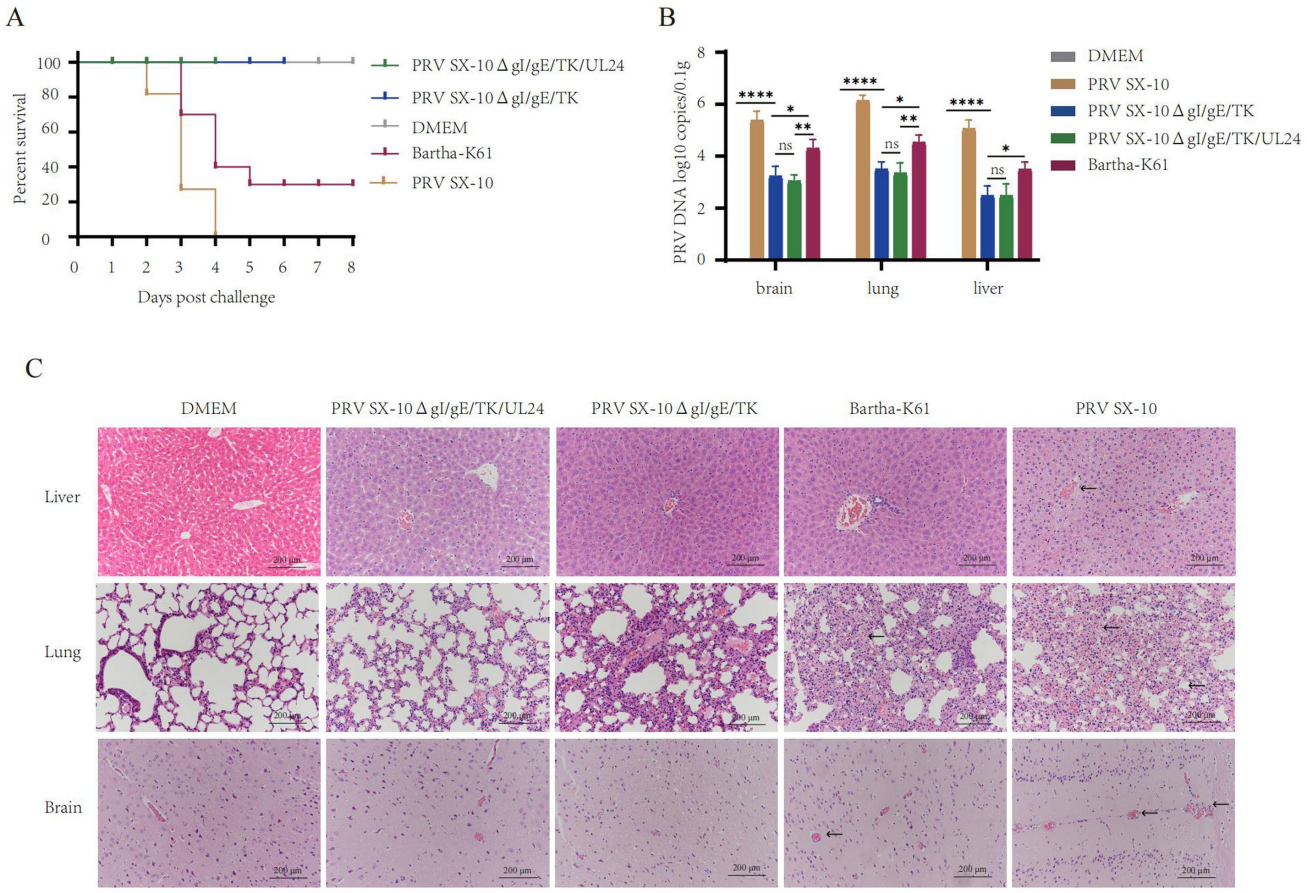
Protective efficacy of recombinant virus strains in mice. **(A)** Survival rate of mice challenged with PRV SX-10 strain, DMEM as a control group. **(B)** Quantification of viral loads in the brain, lungs, and liver post-challenge using qPCR. *p < 0.05, **p < 0.01, ****p < 0.0001, and *ns* as not significant. **(C)** Histopathological analysis of liver, lung, and brain from each group after challenge (HE, 200×). Arrows in the PRV SX-10, PRV SX-10ΔgI/gE/TK, and Bartha-K61 groups indicate locations of lesions.

At 28 dpi, mice in the PRV SX-10ΔgI/gE/TK/UL24, PRV SX-10ΔgI/gE/TK, or Bartha-K61 groups were challenged with 100 μL containing 10^5^ TCID₅₀/mL of PRV SX-10, while the DMEM group served as a control. All mice in the PRV SX-10 group and 70% in the Bartha-K61 group exhibited typical PR symptoms and succumbed to the infection between 3 and 6 days post-challenged (dpc). In contrast, all mice in the PRV SX-10ΔgI/gE/TK/UL24, PRV SX-10ΔgI/gE/TK, and DMEM control groups survived (Figure 5A). Compared to the PRV SX-10ΔgI/gE/TK/UL24 and PRV SX-10ΔgI/gE/TK groups, significant viral genomic presence was detected in the brain, lungs, and liver tissues of mice in the Bartha-K61 and PRV SX-10 groups (Figure 5B). At 14 days dpc, histopathological examinations were conducted on the mice. In the PRV SX-10 and Bartha-K61 groups, mice exhibited markedly enlarged vascular sleeve phenomena in brain tissues, accompanied by neuronophagia. Additionally, these mice developed pulmonary edema, severe hemorrhage, and alveolar destruction in the lungs, as well as hepatocyte degeneration in the liver. In contrast, mice immunized with PRV SX-10ΔgI/gE/TK/UL24 exhibited no discernible histopathological lesions or pathological damage. However, mice in the PRV SX-10ΔgI/gE/TK group exhibited mild inflammatory cell infiltration and congestion in the lungs (Figure 5C).

### Evaluation of safety and immunogenicity of PRV SX-10ΔgI/gE/TK/UL24 in piglets

**Figure 6:**
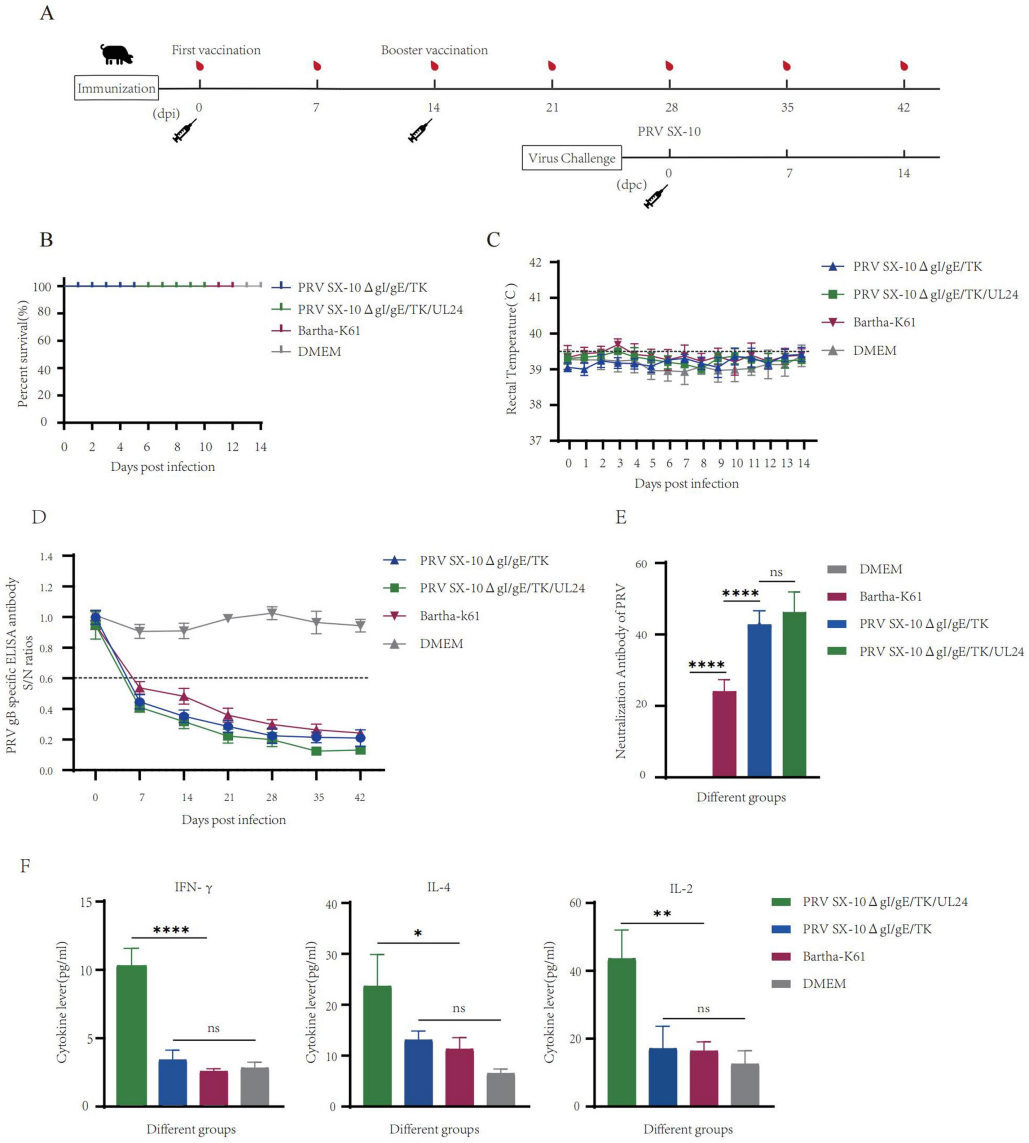
Pathogenicity assessment of the recombinant virus strain PRV SX-10ΔgI/gE/TK/UL24 in piglets. **(A)** Immunization and challenge timeline for piglets. **(B)** Survival rate of piglets immunized with PRV SX-10ΔgI/gE/TK, PRV SX-10ΔgI/gE/TK/UL24, Bartha-K61 strains, DMEM as a control group. **(C)** Rectal temperatures of all piglets measured daily after inoculation. **(D)** Detection of gB-specific antibodies in different groups at various time points. ELISA values of serum samples are expressed as signal-to-noise (S/N) ratios, with S/N ≤ 0.6 considered positive and S/N > 0.6 considered negative. **(E)** Determination of virus-neutralizing antibody titers in serum collected at 28 dpi. **(F)** Detection of cytokine expression levels of IFN-*γ*, IL-2, and IL-4 in the serum of piglets at 28 dpi using ELISA. *p < 0.05, **p < 0.01, ****p < 0.0001, and *ns* as not significant.

To further evaluate the safety profile and immunogenic potential of recombinant virus, piglets were immunized with either PRV SX-10ΔgI/gE/TK/UL24, PRV SX-10ΔgI/gE/TK, Bartha-K61, or DMEM as a control (Figure 6A). At 14 dpi, all piglets in the vaccinated groups remained alive and in good health (Figure 6B). Rectal temperatures were monitored, and no significant differences were observed compared to the DMEM control group. Temperatures consistently remained below 40.0°C in all groups (Figure 6C). Piglets inoculated with PRV SX-10ΔgI/gE/TK/UL24, PRV SX-10ΔgI/gE/TK, or Bartha-K61 strains produced PRV-specific gB antibodies, with titers increasing progressively over time. However, PRV SX-10ΔgI/gE/TK/UL24 produced significantly higher antibody levels than Bartha-K61 (Figure 6D). At 28 dpi, piglets inoculated with PRV SX-10ΔgI/gE/TK/UL24 or PRV SX-10ΔgI/gE/TK strains showed comparable levels of neutralizing antibodies (NAbs) against PRV SX-10. These groups exhibited significantly higher NAb titers than the Bartha-K61-vaccinated cohort (*p* < 0.05) (Figure 6E). Furthermore, at 28 dpi, the group inoculated with PRV SX-10ΔgI/gE/TK/UL24 exhibited significantly higher levels of IFN-*γ* compared to groups inoculated with PRV SX-10ΔgI/gE/TK, Bartha-K61, or DMEM (*p* < 0.01). Serum levels of IL-2 and IL-4 were also significantly elevated in the group inoculated with PRV SX-10ΔgI/gE/TK/UL24 (*p* < 0.05) (Figure 6F).

### PRV SX-10ΔgI/gE/TK/UL24 provide better protection in piglets

**Figure 7:**
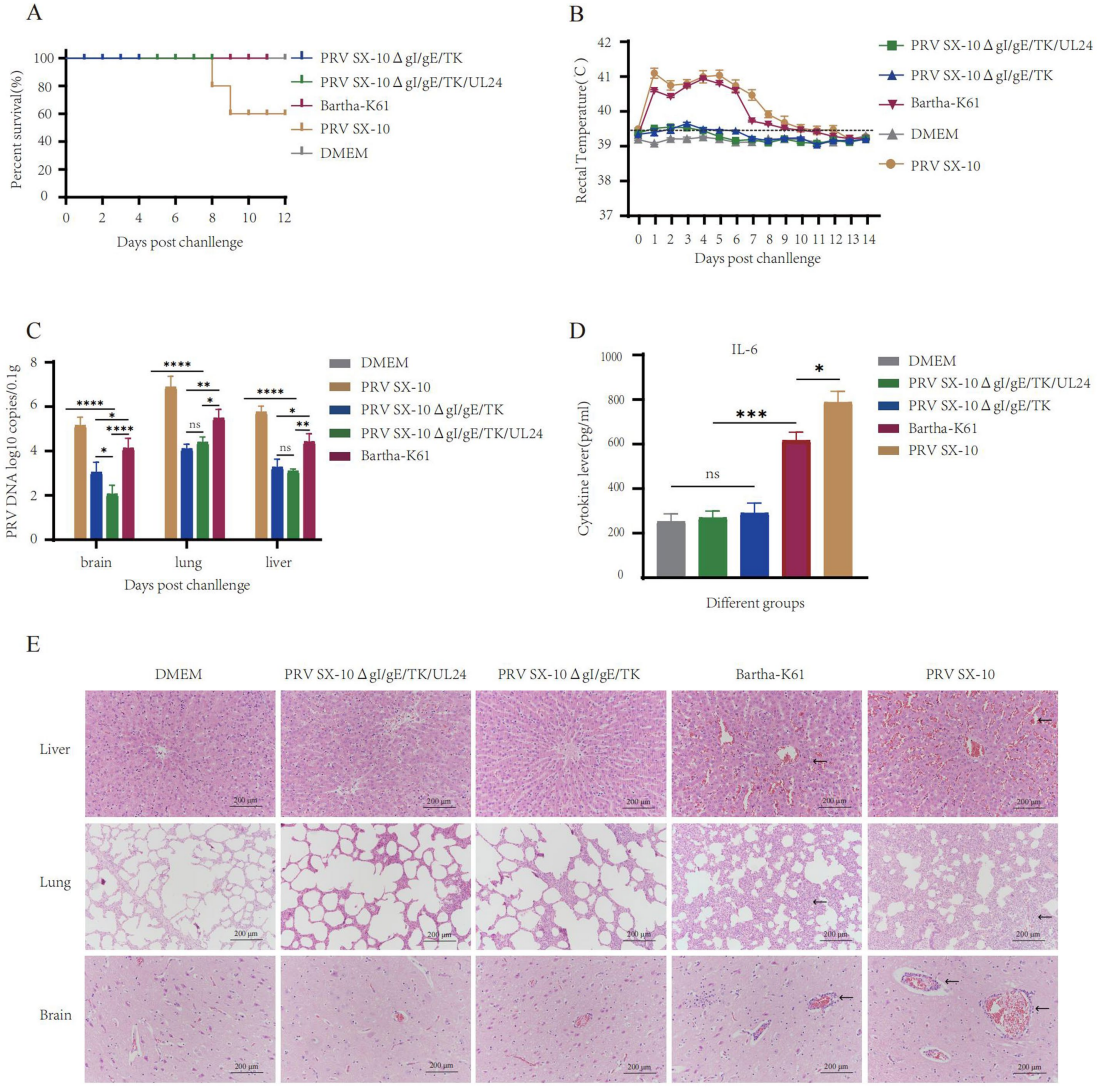
Protective efficacy of immunization with the strain PRV SX-10ΔgI/gE/TK/UL24 against challenge with variant strain PRV SX-10 in piglets. **(A)** Survival rate of piglets challenged with PRV SX-10 DMEM as a control group. **(B)** Measure the rectal temperature of piglets daily after challenge. **(C)** Detection of viral loads in the brain, lungs, and liver after challenge using qPCR. **(D)** Detection of cytokine expression levels of IL-6 in the serum of piglets at 3 dpc using ELISA. *p < 0.05, **p < 0.01, ***p < 0.001, ****p < 0.0001, and *ns* as not significant. **(E)** Histopathological analysis of liver, lung, and brain from each group after challenge (HE, 200×). Arrows in the PRV SX-10 and Bartha-K61 groups indicate locations of lesions.

At 28 dpi, piglets from the PRV SX-10ΔgI/gE/TK/UL24, PRV SX-10ΔgI/gE/TK, and Bartha-K61 test groups were challenged with 10^6^ TCID₅₀/mL of virulent PRV SX-10 strain, except for the DMEM control group. Piglets in the Bartha-K61 and PRV SX-10 groups exhibited typical pseudorabies symptoms, including pruritus and sneezing. Notably, two out of five piglets infected with the PRV SX-10 strain succumbed to the infection within 7–9 days post-inoculation. In contrast, piglets inoculated with PRV SX-10ΔgI/gE/TK/UL24, PRV SX-10ΔgI/gE/TK, Bartha-K61, or DMEM exhibited no discernible clinical symptoms (Figure 7A). For the first 9 days after infection, rectal temperatures in the PRV SX-10 and Bartha-K61 groups surpassed 41.0°C, indicating severe febrile responses (Figure 7B). Viral load quantification indicated low viral presence in the tissues of piglets immunized with the PRV SX-10ΔgI/gE/TK/UL24 and PRV SX-10ΔgI/gE/TK strains, with the former group exhibiting significantly lower titers in brain tissue (*p* < 0.05) (Figure 7C). Cytokine analysis conducted 3 dpc with PRV SX-10 revealed a significant upregulation of IL-6 in the PRV SX-10 and Bartha-K61 groups compared to groups vaccinated with PRV SX-10ΔgI/gE/TK/UL24, PRV SX-10ΔgI/gE/TK, or treated with DMEM (Figure 7D). Histopathological examination revealed severe tissue damage in piglets from the PRV SX-10 group. Affected tissues included lung alveoli with inflammatory infiltration and hemorrhage, brain tissue showing neuronal degeneration, and liver tissue displaying hepatocyte swelling and vascular dilation. Comparable pathological alterations were observed in the Bartha-K61 group. In stark contrast, piglets in the PRV SX-10ΔgI/gE/TK/UL24, PRV SX-10ΔgI/gE/TK, or DMEM groups exhibited no significant histopathological alterations (Figure 7E).

## Discussion

Since 2011, Pseudorabies virus (PRV) variant strains have undergone extensive genomic mutations (He et al., 2018). Despite widespread vaccination with the Bartha-K61 strain, Pseudorabies (PR) outbreaks have persisted (An et al., 2013; Luo et al., 2014; Yu et al., 2014). Immunization with attenuated vaccines remains the fundamental strategy for mitigating PRV outbreaks (Freuling et al., 2017). In this study, a recombinant PRV mutant strain with gI/gE/TK/UL24 deletions was constructed using the CRISPR/Cas9 system. Animals models inoculated with high doses of PRV SX-10ΔgI/gE/TK/UL24 exhibited neither clinical manifestations, such as pyrexia or pruritus, nor gross organ lesions. Notably, piglets inoculated with PRV SX-10ΔgI/gE/TK/UL24 demonstrated significantly lower viral loads in cerebral tissue compared to those inoculated with the PRV SX-10ΔgI/gE/TK vaccine. Furthermore, in murine models, the PRV SX-10ΔgI/gE/TK/UL24 inoculation did not induce significant pulmonary damage, in contrast to the PRV SX-10ΔgI/gE/TK inoculation group. These findings suggest that the PRV SX-10ΔgI/gE/TK/UL24 strain exhibits enhanced safety profiles in both murine and porcine models while effectively controlling infections caused by variant PRV strains.

PRV, a herpesvirus with an extensive genome, presents significant challenges for *in vitro* genetic engineering (Horsburgh et al., 1999; Kamel and El-Sayed, 2019; Thomsen et al., 1987; Yuan et al., 2023). Historically, gene deletion vaccines were primarily constructed using bacterial artificial chromosome (BAC) infectious clones or homologous recombination gene editing techniques (Messerle et al., 1997; Stavropoulos and Strathdee, 1998; Warden et al., 2011). However, these methods were time-consuming, labor-intensive, and suffered from low recombination efficiency, thereby posing significant obstacles for PRV vaccine development and research (Baron et al., 2018; Thomsen et al., 1987). The advent of CRISPR/Cas9, a cutting-edge genome modification tool, has markedly expedited DNA vaccine innovation (Cong and Zhang, 2015; Wei et al., 2024; Yang et al., 2020). CRISPR/Cas9-mediated gene editing of PRV is now widely employed to construct diverse vaccine candidates with targeted deletions, including strains lacking gI/gE/TK/UL13 (Lv et al., 2021), PK/gE/TK (Xu et al., 2022), or TK/gI/gE/11 k/28 k genes (Yan et al., 2021), among others (Deng et al., 2022; Jin et al., 2022; Li et al., 2020). Building upon the research of Zhang Feng team’s on SpCas9 nickase and chimeric guide RNA expression plasmids, the commercialized pX335 gene editing vector was modified (Ran et al., 2013). The vector was engineered to carry dual sgRNA expression cassettes, each driven by a U6 promoter. This adaptation enables simultaneous gene editing at sites on both ends of the target fragment, significantly enhancing the overall efficiency of the gene editing process (Luo et al., 2022). In the present study, two pairs of sgRNA cutting plasmids were designed, targeting both ends of the UL24 and TK/UL24 loci, respectively. Additionally, a CMV-driven EGFP reporter gene expression cassette was constructed to replace the knocked-out fragments. Following five cycles of plaque refinement, the PRV SX-10ΔUL24 and PRV SX-10ΔgI/gE/TK/UL24 virus strains were successfully constructed. This approach further improved the precision of knocking out PRV virulence genes and knocking in the reporter gene.

The primary challenge in PRV prevention and control lies in its capacity to establish permanent latent infection in hosts and its robust evasion capability capability (Brukman and Enquist, 2006; Lamote Jochen et al., 2017; Liu et al., 2021; Thoresen et al., 2021). The UL24 gene of herpesviruses has been demonstrated to modulate multiple immune signaling pathways, contributing to immune evasion of the host’s antiviral response (Gao et al., 2022; Kolb et al., 2016; Xu et al., 2017). Similarly, the PRV UL24 gene exhibits regulatory function in the host’s innate immune response (Chen et al., 2021a; Chen et al., 2021b; Wang et al., 2020). Investigations into the UL24 gene in PRV variant strains revealed that, whether deleted individually or in combination with major virulence genes gI, gE, and TK, a relatively high level of IFN-*β* transcription is observed *in vitro*. This not only reduced immune escape from the host but also induced a stronger immune response, consistent with previous studies (Liu et al., 2021). The UL24 gene knockout virus strain demonstrated normal replication *in vitro*, exhibiting a growth curve comparable to the parental strain. Moreover, the survival rate of mice infected with this strain increased by 20% compared to those infected with the wild-type virus. However, the relatively high mortality rate following PRV UL24 deletion is attributed to the fact that the key virulence gene in PRV remains the TK gene (de Leeuw and van Oirschot, 1985; Kit et al., 1987). Antibody titers in mice immunized with PRV SX-10ΔgI/gE/TK/UL24 were significantly higher than in other immunized groups, and antibody levels in immunized piglets were significantly elevated compared to Bartha-K61 group. Notably, neutralizing antibody levels produced in mice and piglets inoculated with PRV SX-10ΔgI/gE/TK/UL24 and PRV SX-10ΔgI/gE/TK were markedly higher than those in subjects inoculated with Bartha-K61. This findings suggest that PRV SX-10ΔgI/gE/TK/UL24 possesses strong immunogenicity, and the deletion of the UL24 gene May induce a stronger innate immune response. Furthermore, compared to other inoculated groups, levels of cytokines such as IFN-*γ*, IL-2, and IL-4 in the serum of piglets inoculated with PRV SX-10ΔgI/gE/TK/UL24 were significantly elevated. Notably, after PRV SX-10 challenge, IL-6 levels in piglets inoculated with PRV SX-10ΔgI/gE/TK/UL24 and PRV SX-10ΔgI/gE/TK were significantly lower than in piglets inoculated with Bartha-K61, indicating a reduced inflammatory response.

In conclusion, the engineered PRV strain SX-10ΔgI/gE/TK/UL24 demonstrates robust replication capacity with significantly attenuated virulence and enhanced immunogenicity in both murine and porcine models. This recombinant strain represents a substantial advancement in PRV vaccine development, effectively combining safety, immunogenicity, and protective efficacy. The insights gained from this study provide a valuable framework for the development of more efficacious PRV vaccines, crucial for safeguarding the swine industry against the evolving threat of emergent PRV strains. Further research is warranted to fully evaluate the potential of this promising vaccine candidate in field conditions.

## Materials and methods

### Cells, viruses, and animals

Both BHK-21 and PK-15 cells were grown in DMEM with added serum and antibiotics. Cells were kept at 37°C in a special incubator with controlled humidity and CO_2_ levels. The PRV SX-10ΔgI/gE strain was previously constructed and maintained in our laboratory (Luo et al., 2022). The PRV SX-10 variant strain, isolated from a pig farm in Shaanxi, was used for neutralizing antibody tests and challenge protection experiments. Six-week-old female Kunming mice were obtained from Chengdu Dossy Experimental Animals Co., Ltd. (Chengdu, China). Four-week-old male American Landrace piglets, weighing 7–9 kg, seronegative for PRRSV, PCV2, CSFV, PPV, and PRV, were purchased from Shaanxi Shunxin Pig Breeding Co., Ltd. (Shaanxi, China).

### Design and assembly of transfer and sgRNA plasmids

To generate a PRV mutant deficient in gI, gE, TK, and UL24, we constructed a donor plasmid with UL24/TK deletions based on the PRV SX-10ΔgI/gE strain. Homology arms of 1,150 bp and 1,398 bp were amplified using primers UL25 LA-F/R and UL22 RA-F/R, respectively, with the SX-10 viral genome serving as the PCR template. These homology arms were subsequently inserted flanking a CMV promoter-driven EGFP reporter gene, resulting in the plasmid designated pUC57-UL25LA-CMV-EGFP-polyA-UL22RA. A similar method was used to construct a plasmid with only the UL24 deletion, which was named pUC57-UL25LA-CMV-EGFP-polyA-TKRA. To facilitate targeted deletions of the UL24/TK and UL24 genes, we employed the CRISPR tool^1^ to design an sgRNA system. The dual sgRNAs were then individually cloned into a modified pX335 vector using BbsI and BsaI restriction sites. Primer and sgRNA sequences used are listed in Table 1.

**Table 1:** PCR primer.

Target gene	Primer sequence (5′-3′)
TK	F-CGCACTCTGTTCGACACGGA
	R-GCTGATGTCCCCGACGATGA
gI/gE	F-AGCCCAAGATGACGTTGGCC
	R-AGGTCGCCGTTGAGGTCATC
UL24	F-AGCGGCACGTCTTGAGCTC
	R-AACAGAGTGCGCCAGTACGC
EGFP	F-ATGGTGAGCAAGGGCGAGGAG
	R-TTACTTGTACAGCTCGTCCATGC
mCherry	F-ATGGTGAGCAAGGGCGAGGAG
	R-CTACTTGTACAGCTCGTCCATG
IFN-β	F-TGCAACCACCACAATTCC
	R-CTGAGAATGCCGAAGATCTG
β-action	F-CTCCTTCCTGGGCATGGA
	R-CGCACTTCATGATCGAGTTGA
gH	F-ACAAGTTCAAGGCCCACATCTAC
	R-GTCYGTGAAGCGGTTCGTGAT
	P-FAM-ACGTCATCGTCACGACC-TARAM
UL25 LA	F-AGCACGCTGTGGCCCTCCAGCGCGTAGC
	R-GCATGCGCGTGCTCGACGTGACGCGCCTGC
UL22 RA	F-CCTCGCCCCTCCCACCCGCGCCGCGGCCGGA
	R-AGGTTGGCCAGGGTGGCGTCCCCGCCGAG
TKRA	F-ATGCGCATCCTCCGGATCTACCTCGACGGC
	R-CACGGACGACGCGGGCATGGTGACGGGCA
sgRNA 1	F-CACCGGTGCTCTTGCCGGTGCCGT
	R-AAACACGGCACCGGCAAGAGCACC
sgRNA 2	F-CACCGCGCCTTCACGTCGGAGATG
	R-AAACCATCTCCGACGTGAAGGCGC
sgRNA 3	F-CACCGCCCGGCGACGTACTCGGCG
	R-AAACCGCCGAGTACGTCGCCGGC
SgRNA 4	F-CACCGCGGCACCGGCAAGAGCACCA
	R-AAACTGGTGCTCTTGCCGGTGCCGC

### Generation of recombinant viral strains using CRISPR/Cas9-mediated genome editing

Gene deletion in the virus was achieved by leveraging the host cell’s homology-directed repair (HDR) mechanism and CRISPR/Cas9 technology. BHK-21 cells were cultured in 12-well plates and co-transfected with either pX335 sgRNA1/2-TK/UL24 (2 μg) or pX335 sgRNA3/4-UL24 (2 μg), along with pUC57-UL25LA-CMV-EGFP-polyA-UL22RA (2 μg) or pUC57-UL25LA-CMV-EGFP-polyA-TKRA, using TurboFect transfection reagent as per the manufacturer’s instructions. At 24 h post-transfection, cells were infected with either PRV SX-10ΔgI/gE or PRV SX-10 at a multiplicity of infection (MOI) of 0.01. Upon observation of extensive cytopathic effect (CPE), cells and supernatants were harvested, subjected to multiple freeze–thaw cycles, and used to re-infect fresh cells. These cells were then overlaid with a mixture of 2× DMEM and 1% low-melting agarose. Using an inverted fluorescence microscope, researchers observed the development of plaques exhibiting green and red fluorescence, or solely green fluorescence. These fluorescent plaques underwent multiple rounds of isolation and purification. To confirm the identity of the recombinant viruses, PCR analysis was conducted using the specific primers outlined in Table 1. Finally, the PRV SX-10ΔgI/gE/TK/UL24 and PRV SX-10ΔUL24 deletion viruses were generated via plaque purification.

### *In vitro* characterization of PRV strains: growth kinetics, plaque morphology, and genetic stability

Five PRV strains (PRV SX-10ΔgI/gE/TK/UL24, PRV SX-10ΔgI/gE/TK, Bartha-K61, PRV SX-10, and PRV SX-10ΔUL24) were evaluated for their one-step growth kinetics and plaque morphology in PK-15 cells. For growth kinetics, PK-15 cells cultured in 12-well plates were infected with each virus at a MOI of 0.01. Cell supernatants were harvested at 12, 24, 36, 48, 60, and 72 h post-infection, subjected to three freeze–thaw cycles, and stored at −80°C. Virus titers were determined by TCID_50_ assay and used to construct growth curves for each strain. For plaque assays, PK-15 cells in 12-well plates were exposed to viruses at a MOI of 0.01 for 90 min, then overlaid with DMEM containing 2% FBS and 1% low-melting agarose. At 96 h post-infection, plaques were visualized by crystal violet staining, and their sizes were quantified using ImageJ 2.7 software.^2^ To assess the genetic stability of the different PRV strains, recombinant viruses were serially passaged 21 times in BHK cells. Viral DNA was extracted every five passages, and the TK, gEgI, EGFP, and mCherry genes were amplified using gene-specific primers TK-F/R, gEgI-F/R, EGFP-F/R and mCherry-F/R, respectively (Table 1).

### Ultrastructural analysis of virions by transmission electron microscopy

PK-15 cells were cultured in 6-well plates. When the cell density reached 80%, each well was inoculated with PRV SX-10ΔgI/gE/TK/UL24, PRV SX-10ΔgI/gE/TK, PRV SX-10ΔUL24, and PRV SX-10, respectively, at a MOI of 0.1. The plates were incubated at 37°C in a CO_2_ incubator for 90 min. Following incubation, the supernatant was removed and replaced with DMEM supplemented with 2% FBS. The cultures were then maintained for an additional 24 h. Post-incubation, cells were harvested and sequentially fixed with 0.5 and 0.3% glutaraldehyde solutions. The fixed samples were sent to Chengdu Rilai Biotechnology Co., Ltd. for further processing. Viral ultrastructure was visualized using a JEM-1400plus transmission electron microscope.

### IFN-β response and virulence of PRV UL24 variants in cells and mice

The four PRV strains—SX-10ΔgI/gE/TK/UL24, SX-10ΔgI/gE/TK, SX-10ΔUL24, and SX-10—were used to infect PK-15 cells cultured in 12-well plates at a MOI of 1.0. DMEM was used as a negative control. At 12 h post-infection, the supernatant was discarded, and 800 μL of TRNzol Universal reagent (Tiangen Biotech, Beijing, China) was added to each well to lyse the cells and extract total RNA. Subsequently, the extracted total RNA was reverse transcribed into cDNA using a reverse transcription kit (Genestar, Beijing, China) according to the manufacturer’s instructions. RT-qPCR was performed on the obtained cDNA using SYBR reagent (DiNing Biotechnology, Beijing, China) to measure the expression levels of β-actin and IFN-β; the corresponding primers are shown in Table 1. The RT-qPCR was performed using the Tianlong 988 Real-Time PCR System (Tianlong Science & Technology, Shaanxi, China) with the following cycling conditions: initial denaturation at 95°C for 2 min, followed by 40 cycles of 95°C for 15 s, 60°C for 30 s, and 72°C for 30 s. A melting curve analysis was included to verify the specificity of the reaction.

Twenty-five mice were randomly allocated into three groups. Mice were intramuscularly administered 100 μL of either PRV SX-10ΔUL24 (*n* = 10) or PRV SX-10 (*n* = 10) with a virus doses of 10^5^ TCID₅₀/mL, except for the DMEM control group (*n* = 5). The inoculation method and dosage were based on previous studies conducted by our research group (Luo et al., 2022). Clinical signs (including pruritus or scratching) and mortality were monitored and recorded daily to assess the pathogenicity of the PRV SX-10ΔUL24 strain.

### *In vivo* assessment of pathogenicity and immunogenicity in a murine model

Six-week-old female KM mice were randomly allocated into six groups (*n* = 10 per group). Mice were intramuscularly administered 100 μL containing 10^5^ TCID₅₀/mL of PRV SX-10ΔgI/gE/TK/UL24, PRV SX-10ΔgI/gE/TK, Bartha-K61, or PRV SX-10, while the DMEM group served as a control. Clinical signs and survival rates were monitored daily. The clinical signs of PRV infection in mice were primarily observed through the following symptoms: pruritus, scratching and biting of the infected area, tachypnea, circling behavior in some cases, ocular inflammation in others, and in certain instances, sudden death without prior symptoms. At 14 dpi, mice received a second immunization. Serum samples were collected from the retro-orbital sinus of each mouse at designated time points (7, 14, 21, and 28 dpi) for the assessment of PRV-gB-specific antibody levels and neutralizing antibody titers (NAbs). It is noteworthy that the sample collection procedure should not interfere with the normal physiological functions of the mice.

At 28 dpi, all immunized groups, except for the PRV SX-10 and DMEM groups, were challenged intramuscularly (I.M.) with 100 μL 10^5^ TCID₅₀/mL of PRV SX-10. At 14 dpc, all mice were euthanized and necropsied. Brain, lung, and liver samples were collected for viral load determination and histopathological analysis.

### Evaluation of vaccine efficacy and protective immunity against PRV challenge in piglets

Four-week-old weaned piglets, seronegative for PRRSV, PCV2, CSFV, PPV, and PRV, were randomly allocated into five groups (*n* = 5 per group) and housed separately. Piglets in immunization groups 1–3 were intramuscularly inoculated with 2 mL (10^6^ TCID_50_/mL) of PRV SX-10ΔgI/gE/TK/UL24, PRV SX-10ΔgI/gE/TK, or Bartha-K61, respectively. The positive control group (group 4) received 2 mL (10^6^ TCID_50_/mL) of PRV SX-10, while the negative control group (group 5) received 2 mL of DMEM. The inoculation method and dosage for PRV infection in piglets were determined based on previous studies (Lv et al., 2021; Sun et al., 2022; Zhao et al., 2023). At 14 dpi, groups 1–3 received a booster immunization. Serum samples were collected from the anterior vena cava of piglets at specified time points (7, 14, 21, and 28 dpi or 3 dpc) for the assessment of PRV-gB-specific antibodies, neutralizing antibody titers, and cytokine levels.

At 28 dpi, groups 1 through 4 were challenged with 2 mL of PRV SX-10 at 10^6^ TCID₅₀/mL. Piglets were monitored daily for 14 dpi, clinical signs and rectal temperatures were recorded. The primary clinical signs of PRV infection in piglets include pyrexia, ataxia, tachypnea, diarrhea, and pronounced cutaneous erythema. At 14 dpc, piglets were euthanized and necropsied. Brain, lung, and liver samples were collected to assess viral load and histopathology.

### Serological tests and cytokine assays

Serum samples from mice and piglets were collected at predetermined time points for analysis. PRV-specific gB antibodies in pigs were detected using commercially available ELISA kits (JNT, China) according to the manufacturer’s instructions. For mice, PRV-specific gB antibodies were detected using ELISA kits (Finder Biotech, China), with the secondary antibody in the kit replaced by HRP-conjugated goat anti-mouse IgG (Wuhan) at a 1:1,000 dilution. In addition, serum collected from experimental pigs at 28 dpi or 3 dpc was used to quantify IFN-*γ*, IL-2, IL-4, or IL-6 levels using ELISA kits (Cusabio, Wuhan, China), following the manufacturer’s protocol.

Neutralizing antibodies (NAbs) against PRV SX-10 strains were assessed using a modified version of previously described methods (Yu et al., 2016; He et al., 2019; Gerdts et al., 1999). The serum processing involved multiple steps: initial centrifugation (2,800 rpm, 30 min), followed by heat inactivation (56°C, 30 min). The treated serum underwent twofold serial dilutions before being combined with PRV SX-10 strain (100 TCID_50_/mL). This mixture was then incubated at 37°C for 1 h. Subsequently, 100 μL of this mixture was added to confluent PK-15 cells in 96-well plates and incubated for 1 h. The mixture was then replaced with DMEM containing 2% FBS, and the culture was incubated at 37°C under 5% CO_2_ for 4 days. Neutralizing antibody titers were calculated using the Reed-Muench method.

### Quantification of viral load in tissues

Mice and piglets were euthanized at designated time points, and fresh samples were promptly collected and immediately cryopreserved at −80°C for subsequent viral load quantification. One g of tissue sample was weighed and homogenized in a grinding tube containing 1 mL of physiological saline using an ultrasonic homogenizer. Following homogenization, 100 μL of the supernatant was collected and subjected to DNA extraction using a DNA extraction kit (Tiangen Biotech, Beijing, China). Subsequently, real-time quantitative PCR (qPCR) was employed to determine the copy number of the PRV gH gene in brain, liver, and lung tissues. The primers used for amplification are listed in Table 1. A standard curve was established using serial dilutions (10^−4^ to 10^−8^ copies/μL) of the pMD19T-gH positive control plasmid as a template. The qPCR cycling conditions were set as follows: 2 min at 50°C, 2 min at 95°C, followed by 40 cycles of 15 s at 95°C, 30 s at 60°C, and 10 s at 25°C.

### Statistical analysis

Statistical analyses were conducted using one-way ANOVA in GraphPad Prism version 9.0 (GraphPad Software Inc., United States). Significant differences between groups are denoted by asterisks: *p < 0.05, **p < 0.01, and ***p < 0.001. Within each strain, different letters indicate significant differences at a threshold of *p* < 0.05. All data are presented as means ± standard error of the mean (SEM), derived from at least three independent experiments.

## Data Availability

The original contributions presented in the study are included in the article/supplementary material, further inquiries can be directed to the corresponding authors.

## Footnotes

^1^https://zlab.bio/guide-design-resources

^2^https://imagej.nih.gov/ij/
